# Self-help groups as platforms for development: The role of social capital

**DOI:** 10.1016/j.worlddev.2021.105575

**Published:** 2021-10

**Authors:** Carly Nichols

**Affiliations:** University of Iowa, 312 Jessup Hall, Iowa City, IA 52245, United States

**Keywords:** Self-help groups, Behavior-change communication, South Asia, Social capital, Non-governmental organizations, Health and nutrition

## Abstract

Women-led self-help groups (SHGs) are increasingly being utilized as platforms for delivering development activities by funding agencies and governmental bodies. However, there is currently little understanding as to whether SHGs are effective or equitable platforms for delivering health or livelihoods interventions. Social capital is hypothesized as a comparative advantage when utilizing SHGs as development platforms, however the specific mechanisms have yet to be explored. This paper investigates the efficacy and equity of SHGs as platforms for development programs through analyzing 64 interviews and 6 focus group discussions collected from an agriculture and behavior change intervention delivered through SHGs in eastern India. We find that while, theoretically, SHGs are a promising platform for health messaging this is largely dependent on SHG norms of attendance, which itself is closely tied to socioeconomic conditions and social capital. Social capital is important both *within* SHGs as well as between SHGs and the implementing organization. Sites with more mature SHGs had greater economic security allowing more active participation in the intervention than sites with more poverty and young SHGs. The former sites also had greater norms of trust and reciprocity (social capital) with the implementing organization that led them to accept additional interventions. In the latter sites, SHG members had competing demands on their time and less trust in the implementers, making it difficult to attend both SHG meetings and health sessions. We put forth a materialist understanding of social capital formation, where SHG members must have already received substantive benefit from membership for new activities to be successfully incorporated into their agenda. Further, using SHGs as a nutrition message delivery platform should not detract from individual engagement with more vulnerable members of the community.

## Highlights

• Self-help groups (SHGs) are now viewed as promising platforms for multiple development interventions for several reasons.
• Study provides qualitative insights on dynamics of SHG meeting attendance in SHG-led health intervention in India.
• Finds meeting regularity and attendance are barriers to wide coverage, particularly for most vulnerable women.
• Social capital developed between SHGs and sponsor organization is critical to successfully layer health interventions.
• Social capital between SHG and sponsoring organization is developed through benefits received from past association.

## 1 Introduction

Self-help groups (SHGs), groups of 8–20 women engaged in saving and lending activities, have come to dominate the development landscape, particularly in South Asia (Brody et al., 2017, Jakimow and Kilby, 2006). SHGs are formed for a variety of reasons, yet their primary purpose has been to economically empower women and communities through saving/lending activities and bank-linkage programs to access larger pools of capital (Gugerty et al., 2019; GOI, 2019). In India, the central government has catalyzed the spread of SHGs since 2011 through the National Rural Livelihood Mission (NRLM). NRLM’s mandate is to enroll one woman from every poor household into an SHG. By May 2019, the program had mobilized nearly 60 million women in close to 6 million SHGs (GOI 2019). Through taking the SHG concept to scale, the Indian government along with non-governmental organizations (NGOs) and funding agencies,^1^ have increasingly sought to ‘deliver development’ through SHGs by layering thematic interventions onto core saving and lending activities.

Utilizing SHGs as platforms for development programs is attractive to governments and donors for multiple reasons. Foremost, are promises of greater cost-effectiveness and efficiency if information is transmitted to groups rather than individuals. There is also potential for ‘economies of scope’, where development agencies can deliver multiple programs through one intervention platform (Gugerty et al., 2019: 133). While the potential cost-savings may drive interest in SHG-routed development, scholarship also argues that the social capital (resources of trust and reciprocity) generated through regular group interactions gives SHGs a comparative advantage over bringing interventions directly to individuals (Gugerty et al., 2019, Kumar et al., 2018). This is because SHGs with high stocks of social capital have been seen to take collective action in order to demand public goods (Sanyal, 2009). Moreover, transmitting information through SHGs might lead to quicker behavior change (whether it be health and nutrition practices, agricultural improvement, or demanding government entitlements) because groups with more social capital can better regulate norms or sanction ‘deviant’ behavior (Erikkson 2011; Gugerty et al., 2019, Sanyal, 2009). SHGs might also work as spaces of learning where women can experiment with practicing new behaviors and support each other when they face challenges. Women’s empowerment^2^ is often also a key goal of SHGs, and researchers have shown SHG participation can lead to modest improvements in economic, political, social, and psychological dimensions of empowerment (Brody et al., 2017). Women that have control over income and autonomy in decision-making have been shown to make decisions beneficial to their children’s wellbeing (van den Bold et al., 2013).

Due to these above-stated reasons, SHGs have rich potential to trigger impacts on agricultural production, women’s empowerment, or maternal and child health and nutrition (MCHN) but numerous gaps in knowledge remain (Biscaye et al., 2014, Brody et al., 2017, Gugerty et al., 2019, Kumar et al., 2018). To date, the evidence base for SHG-led development is quite mixed, with many studies reporting null results and some finding adverse impacts. Kumar et al. (2018:183) find that short project duration and lack of program ‘coverage’ (e.g. either not enough women are SHG members or do participate in the specific intervention) helps explain why many interventions have negligible impact. This insight is critical because either low rates of SHG participation or members’ irregular meeting attendance would potentially signal a fundamental shortcoming in using SHGs as development platforms. While low participation or irregular attendance in SHGs may be driven by structural factors (e.g. no money, lack of time), they also likely relate to social capital – or the norms of trust and reciprocity in social networks and institutions (e.g. see Lahiri-Dutt and Samanta, 2006, Mayoux and Mayoux, 2001, Sabhlok, 2011). Gugerty et al. (2019) finds evidence to question the efficacy of ‘layering’ additional interventions onto SHGs, while calling for more work that examines how individual members might differentially benefit from interventions. They conclude more evidence on group dynamics—such as participation and attendance norms- is necessary to understand whether the purported advantages of using SHGs for development hold true.

This paper, thus, contributes to the evidence base on using SHGs as development platforms through a qualitative analysis of SHG meeting attendance dynamics in two blocks^3^ of eastern India with SHG-led nutrition interventions^4^. We use data collected within an evaluation of the NGO Professional Assistance for Development Action’s (PRADAN) intervention addressing health and nutrition through layering an MCHN behavior change communication (BCC) program onto its existing SHGs. This paper addresses the primary research question:

How do social capital and socioeconomic conditions interact to enable or hinder the use of PRADAN SHGs as platforms to impart health messages in terms of efficacy (e.g. are women receiving the information) and equity (e.g. are more vulnerable individuals being included)?

In addressing this question, we define social capital as norms of trust and reciprocity and consider socioeconomic conditions to include wealth levels as well as caste and gender norms. This study reveals broader insights around the dynamics of ‘layering’ additional programming onto SHG’s core saving/lending work, which is critically important as the NRLM program looks to expand these efforts in the coming decade (Mitra et al., 2020).

The paper proceeds in five parts. Section two provides an overview of the relevant literature on SHGs and social capital. Section three gives details on PRADAN’s core livelihood and microcredit work and their efforts to layer a BCC intervention onto group activities. Section four describes the methods and study sites in Bastar, Chhattisgarh and Purulia, West Bengal. The fifth section consists of findings and is followed by a sixth section with a short discussion and concluding thoughts on policy recommendations.

## 2 Literature review

**Fig. 1:**
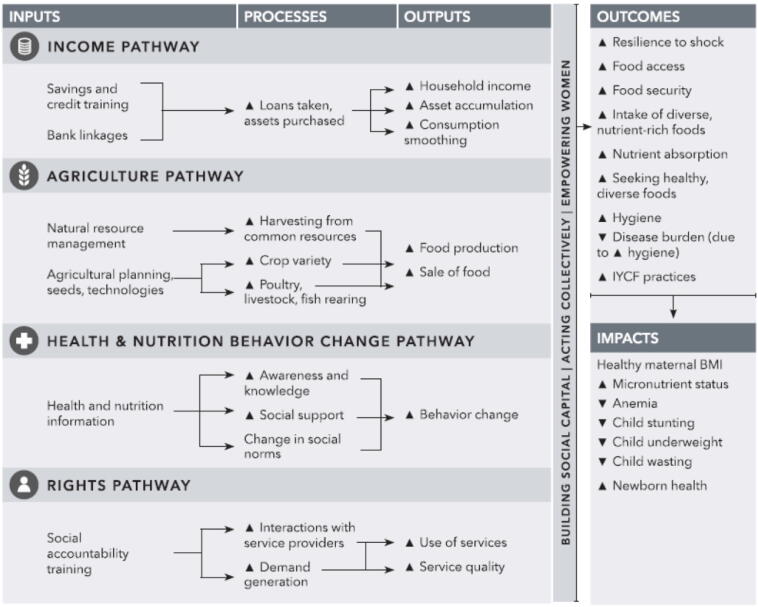
Conceptual pathways from women's groups to maternal and child nutrition (from Kumar et al., 2018).

Recent analyses have conceptualized how SHGs might impact various economic, social, and health related outcomes (Gugerty et al., 2019, Kumar et al., 2018, Kumar et al., 2019, Raghunathan et al., 2019). Kumar et al. (2018) chart four pathways by which different types of SHGs might impact MCHN. The pathways include (i.) income generation, (ii.) agriculture, (iii.) behavior-change communication (BCC), and (iv.) rights awareness. Social capital generation, collective action, and women’s empowerment are identified as cross-cutting pathways that enable impacts on health (see Fig. 1).

Social capital (SC) is often conceptualized as consisting of bonding, bridging, and linking forms (Baland et al., 2008, Szreter and Woolcock, 2004). While bonding SC denotes greater intergroup cohesion, bridging forms refer to social ties outside of one’s social group, and linking forms consist of ties to individuals who wield more power (e.g. political leaders, employers). Mainstream views of social capital thus assert that SHGs harness strong social ties between rural women based on shared ethnic and gender identities (e.g. bonding SC) as a form of collateral to leverage financial loans (e.g. physical capital), build broader networks with other SHGs (e.g. bridging SC), and take collective action to negotiate with more powerful government, private sector, or NGO functionaries (e.g. linking SC) (Rankin, 2002). Kumar et al. (2018:174) reflect this conceptualization to hypothesize that bonding forms of SC, defined as norms of trust and reciprocity, may be harnessed and strengthened through group activities and rituals that translate social ties into SHG best practice and develop into other types of capital formation and collective action to secure public goods (see also Joshi and Rao, 2018, Mitra et al., 2020).

While Kumar et al. (2018) reflect the view that high stocks of bonding social capital are assets within SHG formation, some contend for greater scrutiny of group *heterogeneity* to understand how women may differentially benefit from interventions disseminated through SHGs, based on factors such as education, wealth, or caste, among others (Brody et al., 2017, Gugerty et al., 2019). Here, it is relevant to turn to literature that argues social capital is not *de facto* beneficial, and that in terms of improving the quality of people’s existence it may prove detrimental (Villalonga-Olives & Kawachi, 2017 provide an excellent review). The ‘dark side’ of social capital (Portes & Landolt, 2000) can include both social exclusion as well as excessive demands being made on group members. Reviews argue these adverse outcomes tend to be caused by high levels of bonding capital and the absence of bridging/linking forms of social capital that would allow people to expand beyond group membership and gain access to more resources (Villalonga-Olives & Kawachi, 2017).

Qualitative studies of SHG dynamics have shown that, indeed, sometimes these dark sides play out through excluding women based on caste, education, or wealth (Dahal, 2014, Lahiri-Dutt and Samanta, 2006, Jakimow and Kilby, 2006, Mercer 2002, Sinha et al., 2006) or through creating disappointment when promises of SHG participation are not met (Kumari, 2011, Maclean, 2012). Lahiri-Dutt and Samanta (2006) argue that idealized notions of rural social harmony do not always prevail, and credit local power differentials (based on education, caste, and wealth) for causing SHG breakdowns in West Bengal. While there is little research on whether SHG activities *themselves* (e.g. attending meetings ) create excessive demands on women’s time, some research demonstrates that microfinance loans have ambiguous impacts on women’s time-use depending on how they are utilized and whether men retain control of productive assets (Garikipati, 2012, Zavaleta, 2019). Moreover, much research shows that ‘women-focused’ development projects can create additional labor burdens on women by unduly focusing on them, alone, as the key to economic development thereby adding income-generating activities to responsibility portfolios without helping renegotiate unpaid labor tasks. (Johnston et al., 2018, Rao et al., 2019).

However, another stream of research taking a broader look at institution building argues that projects often underestimate how difficult it is for the extremely poor to devote time to collective action initiatives and it is thus not *de facto* beneficial to focus efforts on increasing social capital under the assumption it will result in physical capital or public goods (Cleaver, 2005, Das, 2004, Portes and Landolt, 2000). Das, 2004, Cleaver, 2005 draw on research from Odisha, India and Tanzania, respectively, to argue socio-economic structural inequities constrain resource-poor people’s agency to engage in social capital-generating institutions (like SHGs) on beneficial terms (also see Baland et al., 2008). Das and Cleaver go further to argue even if rural poor are able to participate in institutions, increased ‘linking’ forms of social capital are not inherently beneficial for the poorest as the benefits of such engagement might not outweigh the opportunity cost of their time.

These more critical perspectives are important to consider when conceptualizing how SHGs might be used as platforms for *additional* development activities beyond core microfinance functions. Little research has examined how social capital may influence SHGs’ willingness to accept other (top-down) development programs ‘layered’ on top of their core work (Gugerty et al., 2019). This is evidenced in Kumar et al.'s (2018) systematic review, which finds that although livelihood SHGs theoretically have the potential to trigger all four sectoral pathways identified in their model –only one intervention successfully did so (Saha et al., 2015).

Saha et al.'s (2015) study is instructive in analyzing how two NGOs with established SHG networks layered a multi-pronged health intervention onto core group activities. They argue the primary factor in program success was that implementing NGOs had “good reputations” due to their long association in the area, and thus had generated enough social capital to gain community trust (Saha et al., 2015: 1516). Moreover, the most active participants were those who had already received monetary and non-monetary benefits from associating with the organization. In the authors’ analysis, when layering additional interventions, *linking* forms of social capital were more important than high stocks of bonding social capital within the SHG, as suggested in Kumar et al. (2018). Moreover, Saha et al. (2015) also implicitly evidence Das, 2004, Cleaver, 2005 more materialist understanding of linking social capital, where it develops through material gains for individuals and groups rather than through institutional interaction.

**Fig. 2:**
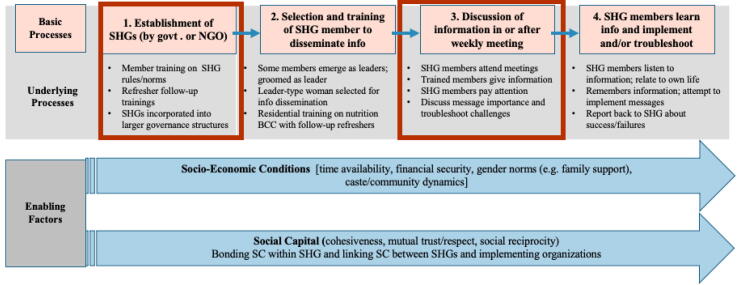
Implementation framework.

With these insights in mind, we build on Kumar et al.'s (2018) conceptual pathways, through putting forth a complementary model that runs parallel to their theory of change. The model depicts the implementation processes necessary to layer interventions onto SHGs, which include: (i.) SHG group establishment, (ii.) selecting and training SHG members to disseminate information (iii.) discussing information in weekly SHG meetings, and (iv.) women implementing this information in their lives or troubleshooting problems in subsequent meetings (see Fig. 2). Similar to Kumar et al.'s (2018) framework, these processes are crosscut by certain enabling conditions, which consist of women’s socio-economic conditions as well as social capital.

We take this framework as a guide to examine our research question on the role social capital and socio-economic conditions play in enabling or hindering layering health/nutrition interventions onto SHGs in terms of efficacy and equity.

In terms of initial groups formation (domain 1), one view posits that bonding forms of social capital are strengthened through regular group interactions to enable collective action and the development of other types of social and physical capital (Kumar et al., 2018). Yet others contend that socio-economic barriers may preclude the most marginal from participating in social capital generating institutions in the first place (Das, 2004). Thus, there are debates as to whether strong social capital within the SHG (bonding) or with the sponsoring agency (linking) may be able to compensate for potential social and economic barriers to participation in SHG meetings and activities.

In terms of layering additional interventions onto groups (domain 3), we hypothesize to see similar dynamics between social capital and socio-economic conditions, where the more marginal have barriers to participation unless they are specifically pulled in through social bonds. Distilling insights from Saha et al. (2015), we hypothesize that reputation and levels of trust/reciprocity between the implementing agency and the SHGs are critical, and that this linking social capital will be formed through actual material gains SHG members have received from group association, in addition to routinized interactions with institutional actors.

Within both domains, literature examining social capital’s ‘dark side’ suggests attention must be paid as to whether social capital results in identity-based exclusions or excessive demands made on group members. Moreover, while some find that bridging/linking forms of social capital may counter against exclusion and excessive in-group demands, others contend that bridging/linking forms of social capital also cannot be viewed as *de facto* beneficial because poor people may feel compelled to enter these relations on terms unfavorable to them. Thus, in analyzing data, particular care was taken to identify not just nuances of how social capital and socioeconomic factors interacted in SHG-led development, but also any evidence of adverse outcomes.

## 3 Study Background: PRADAN SHGs and Facilitating Action Against Malnutrition

PRADAN has been operating across low-caste and indigenous peoples (*adivasi)* areas for the last three decades. PRADAN SHGs are formed for saving and lending activities where women make small weekly deposits (e.g. 10 Indian Rupees, or 15 US cents) into a group account, which enables them to take reasonable interest loans, and become connected to banks and governmental organizations to leverage additional benefits. SHG initiation is centered around saving and lending as an “entry point” to build social capital and trust among women and to provide immediate benefits (in the form of loans), so they may then undertake larger socio-political and economic collective actions. To catalyze these larger transformative actions, PRADAN has also federated SHGs into women’s institutions at village, *panchayat,*^5^ and block levels.

PRADAN recently worked to layer a health promotion and nutrition BCC program onto its SHG institutions in eight block sites. PRADAN partnered with Public Health Resource Society (PHRS), who has expertise in MCHN to implement a BCC program known as Facilitating Action Against Malnutrition (FAAM). FAAM’s main goal was to leverage PRADAN’s large SHG network to increase awareness among women on the underlying causes and practices associated with malnutrition and to trigger a set of actions among women to reduce malnutrition and anemia (Prasad, 2016).

To achieve this goal, PRADAN’s existing block teams (consisting of 4–6 young professionals) were supplemented with a PHRS public nutrition professional, and a team of 4–5 community nutrition mentors (hereafter known as “mentors”), who were paid a monthly salary of approximately Rs 6000 (approximately USD 83). Teams undertook a community needs assessment to understand the relevant health challenges and their bottlenecks. FAAM was then introduced to SHG leaders in the block-federation and at the village level. SHG members, in collaboration with PRADAN, recruited a cohort of village-level SHG women to serve as nutrition volunteers and carry the nutrition intervention to the SHGs in their village hamlet.

The primary medium to transact the nutrition agenda was a set of nine perspective-building micromodules around key issues such as early marriage, infant and young child feeding (IYCF) practices, and dietary diversity. Each micromodule consisted of a story encapsulating health and nutrition messages and key action points intended to promote healthy behaviors among SHG members. Nutrition volunteers and mentors received instruction on how to deliver each micromodule in 3-day residential trainings, and supplemented with refresher trainings. Nutrition workers were trained to communicate to women that nutrition and basic preventative health practices (e.g. handwashing) would benefit them in the long run. For example, workers would ask how much women spent on healthcare, and then explain healthy nutrition practices would decrease health expenses. If delivered in its entirety, each micromodule lasted approximately 1–2 h and was intended to take place approximately one time per month at the weekly SHG meeting where women would already be meeting for savings. Mentors were tasked with supporting the nutrition volunteers in delivering micromodules.

Impact evaluation data collected at FAAM’s midline (November 2017- January 2018) showed that while the large majority of SHGs (>80%) in intervention villages had participated in the four micromodules delivered at the time, and mentors and nutrition volunteers were proficient in message communication, only 40% of SHG respondents in the intervention reported hearing the most popular FAAM message on dietary diversity^6^. Thus, this present study was conceptualized to explore more nuanced factors within the implementation process as a way to unpack, why, despite high transaction rates and knowledgeable implementation actors, less than half of SHG respondents reported hearing messages.

## 4 Methods and Study Sites

**Fig. 3:**
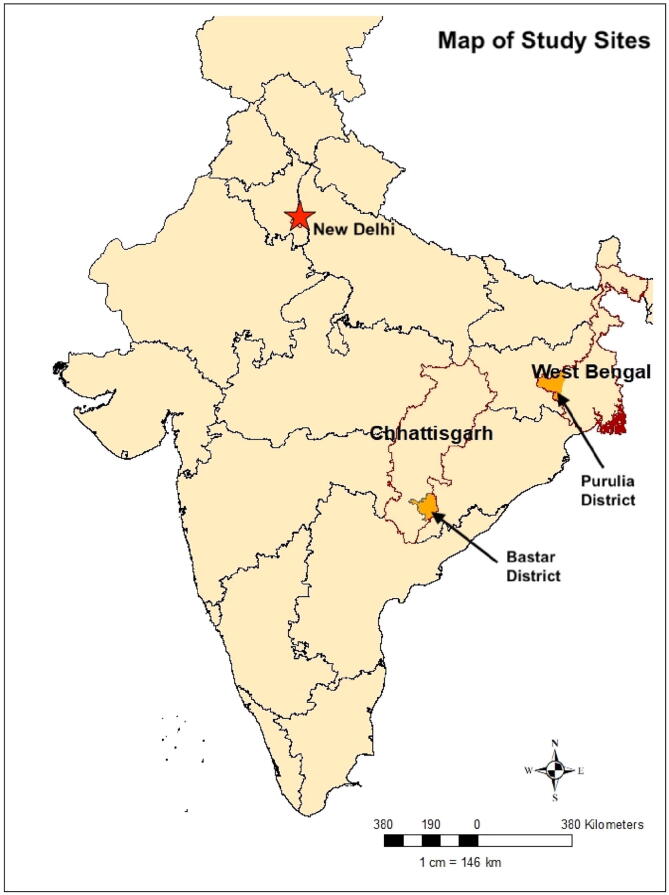
Map of study sites.

Of PRADAN-PHRS’s 8 pilot block sites, we selected blocks^7^ in Bastar District, Chhattisgarh and Purulia District, West Bengal for this qualitative study. (see Fig. 3) These sites were purposefully chosen to have maximum variability in our sample based on both program evaluation data and characteristics of PRADAN’s engagement. They were also selected because neither were pilot sites with earlier exposure and they had maintained fidelity to the implementation plan described above.

SHG respondents in Purulia had the highest levels of recall around FAAM health and nutrition messages compared to other sites (for example, 63% of midline respondents accurately recalled FAAM’s dietary diversity message, compared to 36% in Bastar and 40% averaged across all sites). PRADAN has worked for 18 years in Purulia, making it one of its oldest, most established sites. Similarly, the block-level federation of SHG women was formed 11 years prior, and has regular monthly meetings where representatives from each village-level SHG organization gather. Overall, SHGs have accumulated greater savings than other sites, and also engaged in livelihood improvements with many households growing improved rice varieties and vegetable cash crops. Finally, their socioeconomic demographics are unique in that it is predominantly low-caste Hindus (called ‘other backward castes’ or OBCs in India) with higher literacy rates than other PRADAN sites. (see Table 1). Noteworthy, however, is the large jump in SHGs the Purulia block team has managed over the last 3 years where the SHGs under their guidance more than doubled from approximately 550 SHGs to nearly 1400 as of 2019.

**Table 1:** Block Level Census Statistics (2011 Census of India).

	Purulia District Block-level data		Bastar District Block-level data	
	Total	Female	Total	Female
Population	137,143	67,048	79,360	40,389
Literacy Rate	66.2%	43.8%	38.3%	24.8%
Scheduled Caste	12.4%	12.3%	0.3%	0.3%
Scheduled Tribe	11.4%	11.4%	82.9%	83.2%

Bastar district was selected because at 8 years old, it is one of PRADAN’s newer field sites, and has also rapidly grown its SHG network over the last 3 years as the implementing agency for NRLM. As an NRLM partner they receive government funds to help fulfil the government’s mission to ‘saturate’ blocks with SHGs by enrolling a woman from every household^8^. Bastar is a poorer and more remote area than Purulia district and is heavily dominated by Gond and Halbi tribal groups, also known as *adivasis*. Although Naxal activity has ceased in Bastar over the last 5–10 years, Bastar has historically been a Naxal stronghold, and thus a site of political instability and uncertainty as Naxal rebel groups clashed with government forces. Whereas many SHG members in Purulia practiced improved agriculture, PRADAN has had less success in promoting improved paddy and cash crops in Bastar. Poor adoption was attributed to the fact that Bastar *adivasis* traditionally were not agriculturalists but forest-dwelling hunters. The research aim of selecting two very different sites was to understand common and divergent enabling factors and barriers of the same program in different environmental and socioeconomic contexts.

Conversations with PRADAN workers were critical, as they had internal debates on the relative pros and cons of using mature groups versus younger groups as platforms. Whereas older groups (e.g. Purulia) may be more disciplined in meeting, and have greater stocks of social capital, they also might lose interest in SHGs as they achieve economic development and have less need for low-interest loans or livelihood training. New groups (e.g. Bastar), on the other hand, may be poorly regimented if they do not understand SHGs’ potential benefits, but they may also have greater enthusiasm due to SHGs relative novelty and the immense utility they may serve. These internal questions also motivated our site selection.

We purposefully selected research participants from four distinct groups (i. PRADAN professionals, ii. mentors, iii. nutrition volunteers, iv. SHG members) using the same strategy across both sites (see Table 2). We interviewed all PRADAN-affiliated block level staff that worked directly with FAAM. This included PRADAN professionals (University-educated young Indians recruited from college campuses) as well as mentors, who had completed secondary education and were recruited locally. Using PRADAN internal monitoring data and in collaboration with block teams we purposefully selected 12 SHG nutrition volunteers from different villages to interview and an additional 8–10 for two focus groups.

**Table 2:** Interviews and FGDs Conducted in Each Study Site.

	Research Participant Subgroups			
	PRADAN Block Professionals	Mentors	SHG Nutrition Volunteers	SHG Members
Total interviews (n = 64)	5	9	24	27
Purulia (n = 33)	2	5	12	14
Bastar (n = 31)	3	4	11	13
Total FGDs Conducted = 6		2	4	
Purulia (n = 16)	–	1	2	–
Bastar (n = 12)	–	1	2	–

We sampled nutrition volunteers to achieve maximum variation in terms of village location, group longevity, and SHG volunteers’ quality of involvement. SHG volunteers had been graded based on the number of micromodules delivered and facilitation skill, so we selected six volunteers from the low-to-medium range and six from the high range. All volunteers were selected from unique villages. Of these 12 volunteers, we selected approximately 6 of them to do additional SHG interviews in their villages. Thus, there were six villages in each block where interviews were conducted with both a nutrition volunteer and also an SHG member.

### 4.1 Data collection and analysis

Data was collected through a combination of semi-structured interviews and focus group discussions (FGD). Interview and FGD guides were prepared and piloted in Purulia district in March 2019 prior to data collection, which took place in May and June 2019. All guides were written in English and then translated by native speakers of the local language.

In each site a three-person team was responsible for the collection, transliteration and analysis of data. The data research manager and interviewer (both local, native language speakers) were trained on the tools and protocols for the research. The interviewer was primarily responsible for data collection, and with informed consent, interviews and focus groups were audio-recorded. In most instances, at least two members of the research team were present for all interviews, with one taking field notes and the other conducting the interview. In the interviews of SHG members and nutrition volunteers we asked what health messages they remembered. For messages they did not recall, we prompted them as to whether it was discussed and, if it was, what they remembered about it. As many women had not heard, or did not remember messages, many interviews were spent exploring whether women felt messages were relevant as well as the dynamics of participation and meeting attendance to better understand variable exposure. We always ended interviews asking respondents what they felt the biggest challenges were related to their health and livelihoods. FGDs were conducted with nutrition volunteers and mentors, respectively, to elicit shared and divergent perspectives on the successes and challenges in delivering the intervention. We prompted participants to share experiences with organizing meetings and delivering micromodules along with broader perspectives on SHG membership. FGDs were useful to understand where there was consensus or disagreement on themes relating to meeting regularity and attendance dynamics.

The data research managers in Purulia and Bastar transcribed and translated interviews and focus group audio recordings. Transcriptions were quality checked by third-party native language speakers. In total 64 interviews and 6 focus groups transcriptions were uploaded to MAXQDA 2018 qualitative data software for analysis. After first reading through the data to take notes on emerging themes, we used a two-cycle inductive coding process that began with developing descriptive codes to parse transcripts into common areas of information (Saldaña, 2013). These sets of commonly coded segments were retrieved and further analyzed using focused thematic coding to describe their variability. For example, while meeting attendance emerged clearly as a dominant descriptive code – communicated by the majority of respondents- women presented different reasons for why women skipped meetings altogether or left them early. While sub-coding we paid attention to not just what was said, but *how* things were said to better contextualize data (Dunn, 2010). For example, if respondents spoke nonchalantly about skipping meetings, we interpreted this to mean such as act was seen as harmless, whereas if the respondents spoke in a distressed manner then it was seen as undesirable/negative. We triangulated themes between the four respondent groups (PRADAN, mentors, volunteers, SHG members) to see if similar information came from each respondent set to establish greater credibility of the data (Patton, 2002). The coding schema was crosschecked by an International Food Policy and Research Institute researcher trained in qualitative methods for accuracy and precision. While themes around meeting and attendance dynamics were discussed by a majority of respondents, themes around the adverse impacts of social capital were found in a smaller set of transcripts (n < 10), yet were communicated by participants from each respondent group. While smaller in number, we considered data points on adverse outcomes analytically important due to the study focus on equity (Saldaña, 2013), and the understanding that potential harm or disparities that may arise from interventions, however small, should be made visible to help prevent their reproduction.

This study has several limitations. Different research teams were used between sites. The Purulia interviewer was experienced, but the Bastar interviewer was a novice and sometimes did not probe systematically. Bastar women also tended to be more reserved in comparison to Purulia women. Several SHG members in Bastar also declined interviews, and the women interviewed tended to be more literate and open to outsiders. Bastar also presented language challenges because the interviewer did not speak Gondi, which many women in interior villages speak. Consequently, Bastar’s SHG sample consisted of women who spoke Halbi (n = 6) or Hindi (n = 7). Mentors supervised the nutrition volunteers, and assisted us with coordinating their interviews. We explained to nutrition volunteers that interviews were anonymous and meant to gather insights to improve future work, yet there was sometimes lingering respondent anxiety in answering questions related to their volunteer work. Relatedly, with any survey/interview asking about normative behaviors (such as SHG meeting attendance) there is a possibility of social desirability bias, where the respondent might not communicate information that they perceive would reflect poorly on them or their village (Dunn, 2010). Nonetheless, for most interviews the research team felt that respondents were forthcoming in their responses. Future research that aims to better ascertain SHG practices might employ participant observation to minimize biases.

## 5 Findings

The findings move through the processes in domains one and three^9^ of the implementation framework (Fig. 2), examining the challenges within each domain as well as the way social capital was drawn upon with both positive and negative effects.

### 5.1 Establishing SHGs and the norm of regular meetings

Forming and ‘norming’ SHG groups is a critical task that must be done well for other interventions to work (domain 1 in Fig. 2). Introductory trainings are given to new SHGs so they understand the norms of weekly meetings, savings, and bookkeeping, however, due to wide variation in socio-economic conditions as well as social capital there is considerable variability in how well SHGs are able to follow such norms.

PRADAN has worked in Purulia for 18 years where it managed a slowly growing number of SHGs. Many of these SHGs had successfully utilized SHG loans for livelihood improvements. As such, many SHGs in Purulia had substantial savings and are regimented in meeting regularly. One Purulia nutrition volunteer explained,

All the *didis*^10^ know about [meeting timings]. They come to the meeting by themselves. Also, all the groups are now having huge savings, so nobody forbids the *didis* to come to the meeting. *(Purulia nutrition volunteer, interview 1a)*

All but two Purulia women reported regular meeting attendance, boasting even during monsoon they would meet in evenings to continue savings. There were regular audits of SHGs to check attendance and financial transactions performed by external community members trained in standard auditing techniques. Groups that performed well were eligible for additional government schemes or loans. Thus, there was a financial benefit incentivizing meeting attendance, and a strong impetus for ‘good groups’ to not lose these benefits.

Conversely, Bastar SHGs had all been formed in the past 2–5 years. They had fewer savings, and were less habituated to meeting weekly than Purulia respondents. Nearly every study village had SHGs that had not met several times in the past month for various reasons. While in one village the SHG had not met for three weeks because they were missing a new register for account tracking *(Bastar nutrition volunteer, interview 5a)*, another village had not met for three weeks because two women had given birth *(Bastar nutrition volunteer, interview 2a)*. In another village we came on the meeting day and the respondent reported the meeting “happens every week,” then quickly qualified they were not meeting that particular day because all the women had gone to the forest to pick a *bhaji (*edible green) to eat and sell at the local market *(Bastar SHG member, interview 8b)*. Bastar nutrition mentors and PRADAN employees reported irregular meetings were their biggest challenge, and it was discouraging to travel to villages for pre-arranged meetings only to find the women had gone for other activities *(Bastar mentor, interview 1c).* Because Bastar groups were relatively young, they often had not been established as reliable in audits and did not have access to as many government benefits. As such, there was less immediate incentive for Bastar women to forgo livelihood or social obligations in order to come to the meeting, which made the opportunity cost for meeting attendance higher than in Purulia.

Other SHGs did not meet regularly because they did not have a literate member to serve as secretary, or their secretary was frequently absent. If they did not have a literate person then it was difficult to maintain the registers for saving/lending activities. In Bastar, there was frequently only one literate woman per SHG who often doubled as the nutrition volunteer. The Bastar mentors explained,

Mentor 1: In my field it is like unless the SHG nutrition volunteer is present, the meeting is not conducted, because she is also the secretary
  Mentor 2: in my work area, it is also the same. The groups don’t meet and it is very difficult there. Only if the *secretary* comes is the meeting conducted. *(Bastar mentor FGD)*

In Purulia, some SHGs would also not meet regularly due to literacy issues. Groups without literate members would rely on a man in the village to do the writing. When the man was absent, they would skip or meet without doing bookkeeping, which put them at risk of defaulting. Thus, across sites, illiteracy among SHG members was a critical mechanism by which exclusions happened since nutrition workers could not disseminate information to groups that did not meet.

Finally, in Bastar, some respondents reported that some groups did not meet regularly because of intimidation by alcohol-abusing men in the village. One mentor said,

In [this one] village the secretary *didi's* husband beat her so therefore she feels scared of coming to the meeting; that is a reason the group is not running well at the place….. that *Dada* always tries to provoke others. *Due to the two or three dadas, there is a fear*. *(Bastar mentor, interview 5c)*

While fear-based barriers to meetings were only in isolated pockets, they were clearly traumatizing to communities, which was reflected in the multiple respondents’ reports of this particular incident.

### 5.2 Individual challenges to meeting attendance

Even when SHG meetings regularly occurred, nearly all respondents reported that not all women attended. The most frequent reason for absences was that women did not have time because they were busy with household or agricultural work or with social obligations (e.g. weddings or funerals). One SHG respondent expounded,

Not all women [attend]. We are farmers. So, we might have some work at the field. It may so happen that some didis get busy with cultivation. When she returns to the village, the meeting might already be over. So, in each meeting 10–12 didis are present—the rest of the didis miss the meeting. *(Purulia SHG member, interview 14b)*

Although *all* women have lots of work, some had much more due to household dynamics (e.g. lack of family support) or economic vulnerability. The poor and vulnerable (e.g. landless or with marginal lands) were often most concerned with securing day-to-day existence, and had difficulty engaging in programs with longer-term benefits (e.g. savings). For example, one landless *adivasi* woman, Goli^11^ said her group was struggling because many women did not attend because they lacked money to deposit. Moreover, the nature of landless women’s work was not conducive to weekly meetings. Goli explained,

[All members] can't come for all the meetings….This is a small village, and there is not much scope for work here, so all of us have to travel quite some distance to work…. …so, it’s very tiring, and sometimes we can't manage it. *(Purulia SHG member, interview 4b)*

While these women understood meetings were beneficial, it was necessary to travel to collect forest goods to ensure their survival. In other Purulia villages, women with less land worked outside the village at brick-making kilns and also frequently missed meetings. In Bastar, many women were daily laborers and worked outside of the village the whole day. Thus, while some women occasionally skipped meetings due to agriculture or household work, those who had to leave the village for labor and livelihoods were consistently unable to attend.

Younger women with small children also frequently missed meetings because they did not have time to go and lacked family support that would enable them to do so. While workloads across sites were gendered, (e.g. women are responsible for care and household work), they could also be generational where the mother-in-law puts more tasks onto the daughter-in-law. In Purulia, four of the five SHG respondents who rarely attended full meetings were young women with small children. These women would maintain SHG membership by sending someone to deposit savings on their behalf. One of these young women, a petite *Adivasi* named Kushma, explained,

I am not able to go. I try, but, I have so much work here. I send my kid to deposit money. I do not have time to speak with my neighbors at our home. I am not able to go. [high voice, showing helplessness] *(Purulia SHG member, interview 10b)*

In a different Purulia village, Sunita, also with a small child, admitted she did not regularly attend meetings. She explained,

I did not go [to the previous meeting], my husband went. I could not go as I have a small kid at home. The meeting happens in the night…I cannot go in the night with the child. We are afraid of elephants in the night. *(Purulia SHG member, interview 1a)*

In Purulia, the practice of sending another person to deposit money on one’s behalf was such a problem, some SHGs levied a fine (5 rupees) if someone skipped a meeting or sent someone to deposit money for them.

Cognizant of the structural barriers to meeting attendance, nutrition mentors were tasked with conducting home visits with particularly vulnerable members. The SHG volunteers were not expected to do formal home visits, but were told to speak about messages in public spaces and to engage absent women if they saw them. Despite these efforts, none of SHG respondents in Purulia or Bastar (inclusive of 2 pregnant woman and 7 young mothers) reported visits from mentors or other members to tell them information. The closest approximation to this was reported by Sunita in Purulia. Sunita said the nutrition volunteer asked her why she was not attending meetings, and that she responded that she was occupied with her child and could not bring them to the meeting in the sun. While the nutrition volunteer inquired to Sunita’s whereabouts, she did not relay health information from the meeting. Sunita had briefly heard some of the more popular messages from neighbors, but had missed most messages. This was surprising as the village nutrition volunteer was highly rated and had a reputation of actively working to mobilize women. Moreover, unlike other young mothers, Sunita was an active SHG member for ten years prior to giving birth. She was quite embedded into the social fabric of the SHG, and yet the messages did not reach her.

Two of Purulia’s other young mother respondents were newer to the village, and less incorporated into social networks. However, these women lived in combined families with their respective mothers-in-law, who were active SHG members. Although their mothers-in-law attended health meetings, neither of the daughters-in-law had heard messages from them, nor been encouraged to attend the meetings. Nutrition workers had inconsistent reports on how mothers-in-law impacted young women’s attendance. One nutrition worker stated mothers-in-law restricted younger women’s access to social networks such as the SHG, while others assumed that mothers-in-law relayed information to these younger women stuck at home.

While the young mothers and pregnant women in both sites had not heard MCNH messages from the SHG, they all heard them from a family member or government worker. While they still had access to basic MCNH information through alternative sources, they were not able to discuss issues in the SHG, receive support for implementation, or troubleshoot challenges.

#### 5.2.1 Social capital’s dark side

Despite the barriers some women faced in attending meetings, there were sometimes disparaging remarks about non-attending women. For example, one quite educated SHG respondent commented, “these women don’t attend the meetings as they don’t understand the problems of the *SHG--* they think that if others go to the meeting, then it is enough" *(Purulia SHG interview, interview 1b*). Others implied that non-attending women were lazy or less educated. Here, we saw a potential dark side of social capital, where women unable to abide by group norms due to socioeconomic marginalization faced discrimination. While there were only approximately five respondents in each site that made discriminatory comments on attendance or meeting regularity, their potential for harm makes them notable.

While most SHGs were socially homogenous in terms of caste^12^ due to anti-*Adivasi* and anti-SC discrimination, there were several mixed-caste SHGs, which sometimes proved problematic. For example, as we interviewed the young, non-attending Purulia SHG member Kushma (an *Adivasi)*, another older SHG member entered the room and angrily protested our choice to interview Kushma since she was not regularly attending meetings. The woman told us if we wanted ‘good information’ we should interview regularly attending women in her hamlet, rather than the *Adivasis* in Kushma’s hamlet. As the woman retreated, we came to find out there were some internal caste-based issues within this group, which was comprised of 10 *Adivasi* women and 5 OBC women from the neighboring hamlet. These women had all been placed in the same SHG because none of the *Adivasi* women were literate. While Kushma stated she did not attend meetings because of workload, it is also possible that these divisions served as a disincentive to attending.

Block professionals and mentors were more sympathetic to the problem of absenteeism. The Purulia mentors expressed that women did not attend meetings because they were new SHG members and had not been properly trained on the benefits to regularly attending SHG meetings. Bastar block professionals also stated introductory trainings were critical to SHGs’ success. They took responsibility for poor group functioning, and stated if women had been better trained on SHG benefits then meetings would happen more routinely. The Bastar team reported that providing so many new SHGs with quality trainings was challenging given their rapid expansion under NRLM, coupled with the tribal language barriers in the district.

In Bastar, there were challenges in providing the institutional support that fostered relationship-building and led women to trust NGO executives’ explanations of SHG benefits. This was also observed in newer areas of Purulia. Critically, although social capital developed through high-quality trainings was lacking, there were also real structural barriers that prevented women from devoting an hour to sit in a meeting. Thus, there was a tension between the PRADAN employees’ narratives that women did not come to meetings because they did not believe in its potential benefits, and women’s own reports that they wanted to come but simply were unable to due to work.

### 5.3 Layering interventions: Social capital is necessary but not sufficient

While there were challenges in simply getting women to attend meetings, there were also challenges in convincing them to stay for additional time to participate in the health modules (domain 3 in Fig. 2). Respondents reported SHG members would often come to deposit money, stay for a bit and then leave early, therefore missing the health portion. In Bastar, mentors were especially distressed with this trend and felt demotivated as a result.

When they [SHG members] just discuss about the savings and do not take health and food seriously, it feels bad, that we have been working hard for so many days and there is no [health] discussion being done. [consensus from others] *(Bastar mentor FGD).*

While this problem was acute in Bastar, Purulia respondents also discussed this frequently. Although, some nutrition volunteers explained women exited if they felt the topic was irrelevant to them, they more frequently stated that SHG members left because they had other work to attend to. One Bastar nutrition volunteer explained the difficulty in getting women to stay for meetings.

But we live in the village. There are difficulties…We would tell – ‘*didi* there is a [health] meeting’, and in return, they would say - ‘you don’t have any work, so you can stay.’ They talk like that. But we would still say – ‘*didi*, do stay for a little while’. So if the *didi* finds time, she would stay for 10–15 min. And she would ask to sign [the participant sheet] and leave *(Bastar nutrition volunteer, interview 7a).*

A Bastar mentor related that this is not an isolated problem, but is present in every village, lamenting.

That is the problem everywhere…[the SHG members] do not give time, if the [nutrition volunteer] wants to tell something they don’t feel like listening to it by sitting there for two minutes. They do not feel that they are getting beneficial information. Once they contribute the money, they feel it’s done. Only those *didi* who are *samajhdar* (wise), say to continue talking. Those who are not *samajhdar*, they would leave. This is the biggest problem in everyone's work area *(Bastar mentor FGD).*

The mentors’ insight that SHG members do not see health information as beneficial was a dominant theme throughout the data. Many respondents echoed this mentor’s assertion that if women were “wise” then they would stay and listen, but that many did not trust that preventative health messages could be beneficial.

Nutrition workers were all trained to utilize motivational tactics to convince women of the utility of good nutrition and health. While these future-oriented communication efforts took place in both sites, Purulia women seemed to trust them more than in Bastar. In Bastar, a more economically marginal district than Purulia, women were more engaged if there was immediate relevance (e.g. she was pregnant or breastfeeding) or if there had been tragic pregnancy-related deaths in their village. Due to economic marginality, Bastar women expressed less ability to prioritize preventative health behaviors over day-to-day livelihood security activities. The situation in Purulia was starkly different and many women spoke excitedly about health messages. In one telling insight, a Purulia nutrition volunteer said every SHG has a few women that intently listen to the health stories. She explained,

"they think that these stories will benefit their children. Even the *dadas* also support them. Now they are going out to other villages to work. Earlier they did not have bank account, or they did not know banking. Now they know." *(Purulia nutrition volunteer, FGD 1)*.

Here, the respondent conflated benefits on health messaging with those of the SHG, more broadly. Her response, corroborated in interviews with mentors and PRADAN staff, implied that women who have *already* seen benefit from the SHG may be more willing to trust this new health-oriented information delivered through the SHG as well as have economic means and social support to enact it.

#### 5.3.1 Linking social capital is generated through material investment in women

The notion that social capital between the NGO and SHG groups was generated through material investment was further evidenced when Purulia mentors explained that villages with sustained PRADAN engagement were more accepting of the nutrition intervention since there was already community trust in PRADAN-led interventions *(Purulia mentor FGD)*. One mentor explained that in his most successful village, PRADAN had already transformed agriculture practices and groups also had high levels of financial transactions. He continued that the weakest village in his field area was quite different, stating,

"[the weak village] had no such [agricultural development] work there. Even PRADAN had not been able to make much of a difference, they had not been able to build up enough trust. Moreover, the people there had not understood the true reason and advantages behind doing the SHGs. That is not the case in [the strong] village. [Nutrition volunteers] there have understood what benefits might be reaped out of the SHGs and these organizations." *(Purulia mentor, interview 3c)*

Thus, there was substantive variability *within* Purulia, where nutrition work was more easily accepted in villages that had benefitted from past PRADAN interventions than in villages where there was less trust and social reciprocity. In Bastar, there were fewer villages where PRADAN had established meaningful relationships or where women had obtained livelihood benefits from SHG membership, thus social capital that linked the SHGs to the implementing organization was altogether lacking. Resultantly, SHG women there were, perhaps, less keen to engage in additional activities beyond the required weekly savings activities (and even that was often skipped).

Additionally, Purulia nutrition volunteers and mentors had been stable over the program length, and many had worked in other roles serving SHG members prior to their nutrition engagement. These nutrition volunteers were well-positioned to draw on bonding forms of social capital to persuade women to stay. Many said they would tell women they had gone to trainings to learn for the village as a way to persuade women to listen to the messages.

NV1: told them that I have gone for training with so much difficulty. It will be great if you attend the meeting.
NV2: I say that “we have given so much time learning all these things. Can you not provide at least one hour to listen to it?” Then they say that they will attend the meeting. Most of the women understand. One or two do not understand. *(Purulia Nutrition Volunteer FGD 2)*

Mentors reported drawing on social reciprocity to maintain good meeting attendance. For example, one Purulia mentor discussed the issue of ensuring women attended the meetings. He said,

Initially, it was tough, but now, over the three years, I have gotten to know all the *didis*. Now if anyone does not come, we go straight to her house to inform her that she should have been present *(Purulia mentor, interview 5c).*

Conversely, in Bastar both high and low-rated nutrition volunteers had a difficult time achieving respect from their village peers. These women had been affiliated with PRADAN for a shorter time and there was less to distinguish them. Bastar respondents reported nutrition volunteers had difficulty mobilizing women to attend meetings, and either a PRADAN executive or mentor were required to get attention. One mentor explained that if it is only the village SHG nutrition volunteer then the SHG women *“just come do the savings and don’t listen patiently to her information.”* She continued,

"the SHG *didis* will make reasons to leave, someone will say they have to get rice, some will say they have to go and get wood. But when [outside people] come then nothing like this happens because [the outside people] tell them, ‘we have come from far to teach you and you have to think-- she is coming from a far place, what is she having to say?’ When we say this the SHG *didis* become little sad and then they say,.. ‘ok *didi*, please tell what you want to!’ So they sit and then they listen." *(Bastar mentor, interview 5c).*

It seemed SHG members in Bastar had not yet developed trust in the volunteer as being a credible source of information, yet when ‘outside’ people came they were compelled to give time since these actors had made sacrifices to come. Bastar mentors and nutrition volunteers also reported exchanging tangible services (e.g. completing writing work for groups) for women’s attention at health meetings.

#### 5.3.2 The dark side of social reciprocity

In both sites, mentors expressed conflicted feelings about invoking social norms of reciprocity to compel participation. One Purulia mentor said that corralling women into meetings caused him great stress. He relayed,

"sometimes we have to force women to participate. This is bad. For example, if a [health] meeting is there I asked '*Didi,* let’s go all to the meeting. *Didi,* you also come.' Somebody might say '*Dada* I cannot go now. I have to clean the rice now' or 'I have to carry the load now'. Or 'I have to buy rice in the evening.' Then I cannot force *didi* to participate in the meeting" *(Purulia mentor, interview 2c).*

The mentor was reflective in these remarks, and it was clear they were in an ethical dilemma where they felt compelled to do their job of disseminating information but also recognized women have time constraints. A Bastar mentor similarly lamented she reprimanded women for going for labor in lieu of attending the SHG meeting, yet knew women needed to earn their living. Both cases exemplify the complicated nature of using SHG meetings as a platform for other activities.

These statements reflect insights that material conditions of poor people’s lives may constrain their agency to engage in social capital-generating institutions. In another example, certain women are selected to attend village-level meetings, which are sometimes located in distant areas. In hot summers this can be problematic. One woman, Manju, said, “*it is a problem [to go to the VO meeting]. But, what to do?! I have to go. Other didis are also coming!”* She continued the meeting was held at 2 pm and in summer it becomes dangerously hot then *(Purulia nutrition volunteer, interview 8a)*. Manju did not want to walk several kilometers in the 100 + degree heat, but because other women were going and she would be reprimanded for non-attendance she felt compelled. In Bastar, another woman relayed a similar experience, where she was returning from an SHG meeting in summer and did not have water so was forced to drink from a polluted river. She said that her group is suffering now because they are not getting regular information from the village meeting, but that she refused to make the journey again.

## 6 Discussion and Conclusion

The vision of SHGs as effective sites for health promotion or other development activities is predicated on several processes within implementation. Most critically, meetings need to regularly happen. SHG meetings are intended to be spaces where women engage in rituals (e.g. opening song/prayer, depositing money, loan repayment) and openly discuss relevant issues. Social capital is hypothetically strengthened through the routinization of these rituals, and a collective is formed that can take action in order to secure other benefits for members. In this assumed ideal, the notion of a “meeting” invokes imagery of a semi-formalized space–time where women all arrive and depart in relative synchronicity to one another and are seen as co-equals.

This study found that SHG meetings often do not proceed as such, and that challenges are tied to both socio-economic barriers and social capital. This analysis highlighted challenges in three points along the implementation pathway: establishment of regular meetings, ensuring all women attended, and ensuring all women would devote extra time to the health module. To recap, due to economic marginalization or low literacy, some groups faced challenges holding weekly meetings. Yet, even when meetings were regularly held, women with young children or daily laborers were at risk of regularly skipping. While safety nets, such as home visits, were designed to reach out to these women, they did not seem effective among our small sample. Moreover, when visits were conducted, the information communicated was abridged. Since modules were participatory and SHGs seen as spaces of social experimentation, missing discussions diminished SHGs' supposed advantages as development platforms. Finally, our analysis found bonding social capital also has a dark side, where some economically or socially (e.g. caste-based) marginalized non-attending members faced discrimination, which sometimes fueled these local tensions. In areas with strong social capital there were also instances of women feeling socially compelled to participate despite this working against their immediate best interest.

We draw two conclusions from this analysis. First, SHG meetings are not standardized blocks of space–time where women automatically gather weekly. The unpredictability within diversified livelihood strategies, along with differing socio-economic conditions, meant that unless women had economic means and social support to attend weekly meetings, attendance was not a priority (especially if relationships with PRADAN staff were weak). Second, women did not automatically accept additional interventions layered onto core saving activities, and sometimes they protested these. However, areas where women had received benefit from SHGs were more likely to engage because their socio-economic conditions enabled them to enact preventative health measures and because there were high stocks of trust and reciprocity with the implementing organization (PRADAN). Thus our hypothesis that high levels of linking social capital are necessary for layering activities, and that such social capital is formed through material investment in SHGs by implementers holds true in this analysis. While this study focused on PRADAN SHGs, the findings are broadly applicable to other livelihood-focused SHGs in South Asia that are formed and maintained by an external organization (e.g. either a governmental or non-governmental agency).

This paper makes two contributions to refine the ways social capital is conceptualized to operate within SHG-based development programs. First, while bonding social capital is conceptualized as central to effective SHG formation and success there has been less emphasis on the role of *linking* social capital between implementers (either NGO or government) and community members. We observe linking social capital to be central in both effective group formation and especially in the layering of additional interventions. Moreover, we observe a *materialist understanding of social capita*l, where women who had received benefits from SHG membership (e.g. loans, agricultural inputs, mentorship) have trust in the institution to potentially generate additional benefits, and also a social obligation to give NGO affiliates (including SHG nutrition volunteers) respect. Thus, where PRADAN had successfully intervened in livelihoods, women were more likely to engage than in areas without previous successful interventions. This builds on and further conceptualizes Gugerty et al. (2019) insight that layering interventions onto SHG platforms might only be successful under certain conditions (e.g. group purpose, meeting frequency, governance). In this study we find that additional programming was more successfully layered within SHGs that also had reported improved socioeconomic conditions as a result of membership. Second, while the SHG literature continues to conceptualize bonding social capital as catalytic to development impact, our data suggests there needs to be greater cognizance of its potential for exclusions and excessive demands on group members. While linking social capital has been seen to allay these negative impacts, we argue linking social capital can also produce ethical dilemmas for implementers conflicted about compelling women with other immediate needs to participate. Though the extent of adverse impacts may be small, it is important programs are cognizant of them since they may exacerbate inequities.

While a detailed analysis of how SHG-based health promotion might successfully proceed from start to finish is beyond the scope of this analysis, we do provide three policy recommendations to guide SHG-routed development. First, development interventions aimed at SHGs that go beyond their core functions need to assess NGO social capital and whether women have achieved initial benefits from SHG involvement. This seems particularly true for information-based interventions rather than ones that offer financial or material benefits. Because not having literate members was a key reason for skipping meetings, SHG-sponsoring organizations might consider greater initial investments in human capital and basic literacy. This would allow groups to function more autonomously, and generate linking forms of social capital NGOs can draw on as they diversify projects. Second, group-based interventions will always risk excluding women who are too busy to attend. As these women can often be more vulnerable or socially excluded community members, group-based programs should not detract from efforts to reach such women individually. Third, women’s absences were largely due to their work burdens, and thus more efforts could be taken to work with other community members (e.g. men or mothers-in-law) to negotiate labor roles and make time for women to attend. Finally, because a key goal of SHGs is to empower women and allow them to exercise more agency, care should be taken to not make excessive demands on their time in the name of SHG activities.

## CRediT authorship contribution statement

**Carly Nichols:** Conceptualization, Investigation, Methodology, Formal analysis, Writing - original draft, Writing - review & editing.

## Declaration of Competing Interest

The authors declare that they have no known competing financial interests or personal relationships that could have appeared to influence the work reported in this paper.

## Footnotes

1 For example the Bill & Melinda Gates Foundation had 49 individual SHG-oriented investments equaling 328 million USD as of 2017 (BMGF 2017)

2 Defined by Kabeer (1999) as an expansion in ability to make strategic life choices that were previously denied.

3 A block is an administrative unit in India, several blocks comprise a district.

4 Based on the literature reviewed above, we find that meeting attendance is one useful proxy to examine whether SHGs are effective platforms for development, since SHGs with poor or variable attendance would not be viable sites for development programs.

5 A *panchayat* is a small administrative unit in India comprising several villages or one large village

6 In control villages with no nutrition intervention there were still approximately 30% of respondents who reported hearing the message.

7 While we selected community-development block in each district we refer to the blocks by district name to preserve anonymity

8 The ‘saturation’ mandate of NRLM is also reflected in PRADAN teams not directly receiving NRLM funds and is the reason SHG numbers in Purulia have also escalated so rapidly in the recent years.

9 Due to space constraints domains two and four are not thoroughly addressed here but are discussed in forthcoming papers

11 All names are pseudonyms to protect respondent anonymity.

12 Either all Other-Backward Castes (OBCs), all-*Adivasis,* or all Scheduled Castes (e.g. Dalits)

## Footnotes

10 *Didi* and *dada are colloquial terms used to respectfully refer to older women and men in Purulia and Bastar.*
